# Local pelvic irradiation modulates Pharmacokinetics of 5-Fluorouracil in the plasma but not in the Lymphatic System

**DOI:** 10.1186/s12885-015-1344-4

**Published:** 2015-04-26

**Authors:** Chen-Hsi Hsieh, Mei-Ling Hou, Li-Ying Wang, Hung-Chi Tai, Tung-Hu Tsai, Yu-Jen Chen

**Affiliations:** 1 Division of Radiation Oncology, Department of Radiology, Far Eastern Memorial Hospital, Taipei, Taiwan; 2 Institute of Traditional Medicine, National Yang-Ming University, Taipei, Taiwan; 3 Department of Medicine, School of Medicine, National Yang-Ming University, Taipei, Taiwan; 4 Department of Radiation Oncology, Mackay Memorial Hospital, Taipei, Taiwan; 5 Department of Medical Research, Mackay Memorial Hospital, Taipei, Taiwan; 6 Department of Education and Research, Taipei City Hospital, Taipei, Taiwan; 7 School and Graduate Institute of Physical Therapy, College of Medicine, National Taiwan University, Taipei, Taiwan

**Keywords:** 5-Fluorouracil (5-FU), Lymphatic, Pharmacokinetics, Radiotherapy, Rectal cancer

## Abstract

**Background:**

5-fluorouracil (5-FU) is employed to enhance radiotherapy (RT) effect. Here, we evaluated the influence of whole-pelvic irradiation on the pharmacokinetics (PK) of 5-FU in plasma and lymphatic system of rats as the experimental model.

**Methods:**

RT with 2 Gy was delivered to the whole pelvis of Sprague–Dawley rats. 5-FU at 100 mg/kg was intravenously infused 24 hours after radiation. The pharmacokinetics of 5-FU in plasma and lymphatic system were calculated.

**Results:**

RT at 2 Gy reduced the area under the plasma concentration *vs.* time curve and mean residence time of 5-FU by 21.5% and 31.5%, respectively compared with those of non-RT controls. By contrast, RT at 2 Gy increased drug clearances of 5-FU by 28.2% when compared with those of non-RT controls. There was no significant difference in T_1/2_, Cmax and Vss in plasma between both groups. Intriguingly, 5-Fu could be detected in the lymphatic system. In addition, the AUC in 5-FU without and with RT was 3.3-fold and 4.9-fold greater for lymph than for plasma, respectively. Compared with the non-RT group, the RT group showed increase in distribution of 5-FU in the lymphatic system (*p* = 0.001).

**Conclusions:**

The local whole pelvic RT at 2 Gy could modulate systemic PK of 5-FU in plasma of rats and intravenous 5-FU passing into the lymphatic system was proved. The metabolism of 5-FU might be modulated by RT but the distribution of 5-FU from blood circulation to the lymphatic system might not be changed. The RT-PK phenomena in plasma provide references for adjustment of drug administration. Chemotherapy drugs entering the lymphatic system is worthy of further investigation.

## Background

Five-fluorouracil (5-FU) is one of the most commonly used chemotherapeutic agents of concurrent chemoradiation therapy (CCRT) for enhancing radiation therapy (RT) effects [1-4]. Lymph node metastases are common among rectal cancer patients with incidence ranging between 5% and 35% even in T1 and T2 stages [5]. For T3, T4 or node-positive rectal cancer patients, adjuvant CCRT improves the locoregional failure control and overall survival by 10-15% when compared with surgery or adjuvant RT alone [6-8]. Further, neoadjuvant CCRT followed by surgery also improves locoregional control for rectal cancer patients [9]. CCRT reduces the risks of local recurrences and regional lymph node metastases.

Growing evidence shows that irradiation may not only has DNA damage effects but also sends signals to their neighborhood, the so-called as bystander effect [10,11] or longer-range effects such as the abscopal effects [12]. Our recent studies reported that local RT could modulate the systemic pharmacokinetics (PK) of 5-FU with different RT doses in an experimental rat model [13,14] through matrix metalloproteinase-8 (MMP-8) [15].

RT may inevitably damage normal tissue and impair the vascular and lymphatic system which could occur already within l h after irradiation [16], further increasing the vascular permeability [17]. While most of the aforementioned studies reported that RT modulates the PKs of 5FU in the plasma, whether RT modulates PKs in the lymphatic system remains largely unknown. The present study investigates the pharmacokinetics of 5-FU in the plasma and lymphatic system in rats with and without RT. Results thus obtained might provide new insights into the RT-PK phenomena of 5-FU.

## Methods

### Materials and reagents

5-FU and ethyl acetate were purchased from Sigma-Aldrich Chemicals (St. Louis, MO, USA). High-performance liquid chromatography (HPLC)-grade methanol was obtained from Tedia Co., Inc. (Fairfield, OH, USA). Milli-Q grade (Millipore, Bedford, MA, USA) water was used for the preparation of solutions and mobile phases.

### Preparation of standard solutions

The standard stock solution was prepared by dissolving 5-FU in Milli-Q water at a concentration of 1 mg/mL and stored at −20°C. The stock solution was diluted with Milli-Q water to prepare a series of working standard solutions. The calibration curves were generated by spiking standard solutions (10 μL) in blank rat plasma or lymphatic fluid (90 μL) and then extracted by ethyl acetate.

### Instrumentation and High performance liquid chromatography (HPLC) conditions

Chromatographic separation was performed with a Shimadzu system, equipped with a chromatographic pump (LC-20AT), an autosampler (SIL-20 AC), a DGU-20A5 degasser and a photo-diode array detector (SPD-M20A) (Shimadzu, Kyoto, Japan). A LiChroCART RP-18e column (Purospher, 250 × 4 mm; particle size, 5 μm, Merck, Darmstadt, Germany) with a LiChroCART 4–4 guard column was used for separation. The mobile phase comprised 10 mM potassium phosphate-methanol (99:1, v/v, pH 4.6), and the flow rate was set at 1 mL/min. The injection volume was 20 μL. The detection wavelength was set at 266 nm. Under these conditions, the retention time of 5-FU was 5.4 min. The linearity of calibration curves was demonstrated by the good determination coefficients (r^2^) obtained for the regression line.

### Method validation

The method validation assays were carried out according to the currently accepted US Food and Drug Administration (FDA) bioanalytical method validation guidance for specificity, linearity, sensitivity, precision, accuracy and recovery. Standard working solutions were obtained by making appropriate dilutions. The concentrations utilized for the calibration curves were 0.5-100 μg/mL for 5-FU. All linear curves were required to have a coefficient of estimation of at least >0.995. The calibration function was constructed by determining the best-fit of peak area. Both intra- and inter-day variability were determined by quantitating six replicates at linear concentrations using the HPLC-UV method described above on the same day and six consecutive days, respectively. The precision (RSD %) and accuracy (bias %) of the analytical method were calculated for evaluation. The extraction recovery of 5-FU from plasma and lymphatic fluid were determined by comparing the peak area of extracted standard in the biological samples with the peak area of standard spiked in neat mobile phase. The accuracy within the calibration range was evaluated by the nominal concentration (C_nom_) and the mean value of the observed concentrations (C_obs_) as follows: accuracy (bias, %) = [(C_obs_ - C_nom_)/C_nom_] × 100. The precision, relative standard deviation (RSD), was calculated from the observed concentrations as follows: RSD (%) = [standard deviation (SD)/C_obs_] × 100.

### Preparation of animals and samples

Both animals and samples were prepared was according to our previous reports [15]. Briefly, adult male Sprague–Dawley rats (300 ± 20 g body weight) were provided by the Laboratory Animal Center at National Yang-Ming University (Taipei, Taiwan). The surgical and experimental protocols involving animals were reviewed and approved by the Institutional Animal Care and Use Committee of National Yang-Ming University (IACUC number: 1020707). They were housed in a specific pathogen-free environment and had free access to food (Laboratory Rodent Diet 5001, PMI Nutrition International LLC, MO, USA) and water.

For radiotherapy, the rats were anesthetized with pentobarbital sodium (50 mg/kg, i.p.), and were immobilized on a board to undergo computed tomography for simulation of the whole pelvic field. The cranial margin was set at the top of bilateral iliac crest for the whole pelvic field. Conventional radiotherapy was employed to deliver the radiation dose through anterior-posterior (AP) and PA portals. The experimental animals were randomized to control (0 Gy) and 2 Gy groups. Data were obtained from 6 rats in each group.

The reason why using 2 Gy for rats to simulate the relevant dose for daily treatment of human torso is safe and workable has been described in our previous report [13]. Briefly, there was no direct comparison of allometric scaling using whole-pelvic irradiation. Nonetheless, the allometric scaling of the lethal dose (LD50) (Gy) of total-body irradiation for human and rat are 4 Gy and 6.75 Gy, respectively [18]. In view of the moderate difference, 2 Gy is used for rats to simulate the relevant dose for daily treatment of human torso.

Ambre et al. [19] studied the elimination of 5-FU and its metabolites to rats. The results of that study suggested that saturation of the catabolic pathway occurred after intravenous administration of 5-FU at 150 mg/kg. When rats were administered 5-FU at 10, 50, or 100 mg/kg in 2 mL of normal saline by intravenous infusion over a 2-min period via the cannula [20] and the dose-normalized area under the curve (AUC) was significantly higher after administration of 100 mg/kg than of 50 mg/kg or 10 mg/kg. The clinical pharmacokinetics of single doses of 5-FU from 300 to 600 mg/m2, administered as intravenous bolus, had been characterized previously [21]. The formula which is used to dose translation from animal to human: human equivalent dose (HED, mg/kg) = Animal dose (mg/kg) multiply by animal Km/human Km [22]. Furthermore, the recommended volume for intravenous (bolus) administration was 5 mL/kg for rats over a short period of approximately 1 min and the rate should not exceed 3 mL/min for rodents [23]. Based on these studies, we chose 100 mg/kg in 2 mL of normal saline by intravenous infusion over a 2-min period as a feasible 5-FU dose in rats for examination of 5-FU pharmacokinetic parameters.

**Figure 1:**
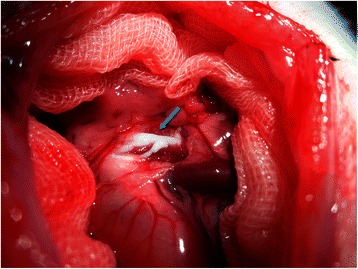
Anatomy of mesenteric lymphatic duct. Light blue arrow indicates the mesenteric lymphatic duct.

For the collection of lymphatic fluid and blood, the rat was given 2 mL of olive oil by oral gavage 30 min before operation to facilitate identification of the lymph duct [24], and then anaesthetized with urethane (1 g/kg) intraperitoneally. Surgical sites were shaved and disinfected with 70% ethanol solution, and polyethylene tubes (PE50) were then implanted into the right jugular vein and left carotid artery for intravenous infusion (normal saline, 2 mL/h) and blood sampling, respectively. The procedure of mesenteric lymph vessel cannulation was performed as previously reported with modification [25]. Briefly, a midline laparotomy was performed from the xyphoid, intestinal mass was displaced with gauze, and the wound was retracted by a 3–0 suture. Mesenteric lymph vessels were easily identified, since they contain white lymph. The mesenteric lymph duct was isolated by teasing away the surrounding tissue with a cotton swab. A small cut was made by a needle, and a silicone tubing (10 cm in length) was inserted into the mesenteric lymph duct (Figure 1). A drop of tissue cement was applied to the hole in the lymph duct to seal it and to fix the cannula in place. The rats were administered 100 mg/kg 5-FU in 2 mL of normal saline by intravenous infusion into the right jugular vein over a 2-min period [20]. Then, 200-μL blood samples were withdrawn from the cannula implanted in the carotid artery into a heparin-rinsed vial at 0, 5, 15, 30, 45, 60, 90, 120, 150, 180, 210 and 240 min. The lymph was collected in heparinized eppendorf tubes at 30-min intervals. The rat was intravenously infused with normal saline at infusion rate of 2 mL per hour throughout the experiment. The samples were immediately centrifuged at 6000 rpm for 10 min.

The resulting plasma and lymphatic fluid (100 μL, respectively) were extracted by ethyl acetate for liquid-liquid extraction. The biological samples were extracted by 1 mL of ethyl acetate twice, vortexed for 5 min, and centrifuged at 13,000 rpm for 10 min. After centrifugation, the upper organic layer containing the ethyl acetate was transferred to a new tube and evaporated to dryness using a vacuum pump. The dried residue was reconstituted with 100 μL of mobile phase. A 20-μL aliquot of the solution was injected to the high-performance liquid chromatography-ultraviolet (HPLC-UV) detection system. The plasma and lymphatic fluid samples were diluted by blank plasma and lymphatic fluid samples at an appropriate ratio before analysis if the 5-FU concentration was exceeded 100 μg/mL.

### Pharmacokinetics

Pharmacokinetic calculations were carried out using a non-compartmental model with the software WinNonlin Standard Edition Version 1.1 (Scientific Consulting Inc., Apex, NC).The areas under a plot of drug concentration versus time curves (AUC) were calculated according to the log linear trapezoidal method. The clearance of the drug (CL) was calculated as follows: CL = dose/AUC. The time required to reduce the drug concentration by half is shown as half-life (T_1/2_) and were expressed as T_1/2_ = 0.693/K, where K is the first-order rate constant. The volume of distribution (Vd) was evaluated as Vd = dose/C0, where C0 is the drug’s plasma concentration. The mean residence time (MRT) was estimated as MRT = AUMC/AUC, where AUMC is the area under the first moment curve. All data are presented as mean ± SEM.

### Statistical methods

The results are presented as mean ± standard error mean (SEM). Differences in actuarial outcomes between the groups were calculated using Student’s *t* test. A *p* value of < 0.05 was considered significant. All analyses were performed using the Statistical Package for the Social Sciences, version 17.0 (IBM Corporation, Armonk, NY, USA).

## Results

### HPLC method validation

Chromatographic conditions, especially analytical columns and mobile phase compositions (concentration of buffer, pH value of the buffer and percentage of the organic modifiers), were optimized to achieve good sensitivity and peak shape, as well as a relatively short run. It was observed that methanol gave a better peak shape than acetonitrile, and was therefore selected as the organic phase. Finally, a mobile phase consisting of methanol- 10 mM KH_2_PO_4_ solution (pH 4.5) was used in the experiment. There was no interference under the present analytical conditions during the retention time of 5-FU which was eluted at 5.1 min. The peak of 5-FU was well separated and there was no endogenous interference in the rat plasma and lymphatic fluid samples. Further, the selectivity was tested by chromatograms of blank plasma and lymphatic fluid spiked with 5-FU standard. Good linearity was achieved in the range of 0.5–50 μg/mL, with all coefficients of correlation greater than 0.995.

The extraction recoveries of 5-FU at low (1 μg/mL), medium (10 μg/mL) and high (100 μg/mL) concentrations in rat plasma versus lymphatic fluid were 59.53 ± 1.83% versus 57.58 ± 1.71%, 56.24 ± 2.24% versus 53.23 ± 5.17%, 56.19 ± 6.65% versus 57.08 ± 2.19%, and with an average of 57.32 ± 4.25% versus 55.96 ± 3.76%, respectively (Table 1).

**Table 1:** **Recovery of 5-FU in rat plasma and lymphatic fluid**

Matrices	Nominal concentration (μg/mL)	Set 1 Peak area	Set 2 Peak area	Recovery (%)
Plasma	1	66301 ± 412	39467 ± 1211	59.53 ± 1.83
	10	632542 ± 4183	355741 ± 14191	56.24 ± 2.24
	100	6463129 ± 22755	3631833 ± 429923	56.19 ± 6.65
	Mean ± SD			57.32 ± 4.25
Lymphatic fluid	1	66301 ± 412	38174 ± 1136	57.58 ± 1.71
	10	632542 ± 4183	336695 ± 32677	53.23 ± 5.17
	100	6463129 ± 22755	3689380 ± 141268	57.08 ± 2.19
	Mean ± SD			55.96 ± 3.76

Data expressed as mean ± SD (n = 6). Recovery calculated as the ratio of the mean peak area of an analyte spiked before extraction (set 2) to the mean peak area of an analyte spiked in the neat mobile phase (set 1) multiplied by 100.

Intra-day and inter-day precision (% RSD) and accuracy (% Bias) were determined by repeated analysis of six lots of biological samples spiked with different concentrations of 5-FU on the same day and six consecutive days, respectively. Precision and accuracy are presented in Table 2. The range of intra-day precision and accuracy in rat plasma versus lymphatic fluid were 0.08% to 18.90% and −5.55% to 2.25% versus 0.02% to 8.67% and −2.74% to 7.60%, respectively. In rat plasma versus lymphatic fluid, the inter-day precision and accuracy ranged from 1.30% to 16.58% and −4.80% to 1.57% versus 0.46% to 5.72% and −0.45% to 3.06%, respectively. The limit of detection (LOD) and quantification (LOQ) of 5-FU in rat plasma and lymphatic fluid were 0.25 and 0.5 μg/mL, respectively.

**Table 2:** **Intra- and Inter-day precision (% RSD) and accuracy (% Bias) of the HPLC-UV method for determination of 5-FU in rat plasma and lymphatic fluid (5 days, 5 replicates per day)**

		Intra-day			Inter-day		
Matrices	Nominal concentration (μg/mL)	Observed concentration (ng/mL)	Precision (% RSD)	Accuracy (% Bias)	Observed concentration (ng/mL)	Precision (% RSD)	Accuracy (% Bias)
Plasma	0.5	0.47 ± 0.09	18.90	−5.55	0.48 ± 0.08	16.58	−4.30
	1	1.02 ± 0.08	7.91	2.25	1.02 ± 0.09	8.81	1.57
	5	4.87 ± 0.24	4.91	−2.58	4.79 ± 0.38	8.00	−4.80
	10	10.15 ± 0.28	2.75	1.52	10.10 ± 0.22	2.13	0.99
	50	49.98 ± 0.04	0.08	−0.04	50.29 ± 0.66	1.30	0.58
Lymphatic	0.5	0.54 ± 0.03	8.67	7.60	0.51 ± 0.03	5.72	1.98
fluid	1	1.06 ± 0.06	5.24	6.30	1.03 ± 0.05	4.63	3.06
	5	4.86 ± 0.19	3.96	−2.74	4.98 ± 0.11	2.24	−0.45
	10	10.03 ± 0.13	1.31	0.30	10.03 ± 0.13	1.27	0.28
	50	50.01 ± 0.01	0.02	0.01	49.91 ± 0.23	0.46	−0.17

Data expressed as mean ± SD.

### Pharmacokinetics of 5-FU in rats

**Figure 2:**
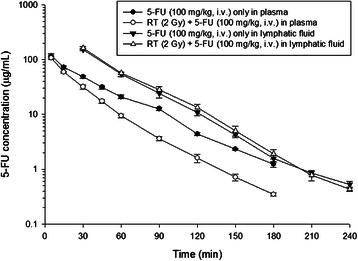
The concentration versus time curves of 5-FU in rat plasma and lymphatic fluid with or without irradiation therapy (RT) after 5-FU administration (100 mg/kg, i.v.). Data are expressed as mean ± SEM (n = 6).

The concentration versus time curves of 5-FU in rat lymphatic fluid and plasma with or without radiation therapy (RT) after 5-FU administration (100 mg/kg, i.v.) to six individual rats for each group are illustrated in Figure 2 and the pharmacokinetic parameters are presented in Table 3. The parameters of PKs of 5-FU in lymphatic fluid with or without irradiation showed no significantly statistical differences. Intriguingly, the AUC of 5-FU in the lymphatic system could be detected suggesting that 5-FU can pass through into the lymphatic system. In addition, the AUC in 5-FU without RT group was 3.3-fold greater for lymph than for plasma. The AUC in 5-FU with RT group was 4.9-fold greater for lymph than for plasma (*p* = 0.001).

**Table 3:** **Estimated pharmacokinetic parameters of 5-FU in rats after 5-FU administration (100 mg/kg, i.v.)**

Parameters	Plasma		Lymphatic fluid	
	Without RT	With RT	Without RT	With RT
AUC (min μg/mL)	4353 ± 257	3108 ± 114*	14240 ± 734	15251 ± 1195
T_1/2_ (min)	28 ± 0.93	26.6 ± 1.67	30.0 ± 6.49	24.4 ± 1.98
Cmax (μg/mL)	119 ± 11.3	102 ± 8.4	153 ± 6.2	163 ± 9.45
CL (mL/min/kg)	23.1 ± 1.41	32.2 ± 1.25*	7.1 ± 0.37	6.73 ± 0.46
MRT (min)	34 ± 1.37	25.9 ± 1.97*	26.3 ± 1.97	27.6 ± 2.38
Vss (mL/kg)	834 ± 63.4	863 ± 50.3	192 ± 20.7	191 ± 26.1

Data expressed as mean ± SEM (n = 6). AUC, area under the concentration versus time curve; T_1/2_, elimination half-life; Cmax, the peak plasma concentration of a drug after administration; CL, total body clearance; MRT, mean residence time; Vss, volume of distribution. *significantly different from without RT group at p < 0.05.

The present results reconfirmed the RT-PK phenomena in the plasma as previously reported that irradiation at 2 Gy markedly reduced the AUC and MRT of 5-FU in rats plasma by 28.6% and 23.8%, respectively. By contrast, irradiation increased the CL of 5-FU by 39.4% when compared with nonirradiated controls. There was no significant difference in T_1/2_, Cmax and Vss between both groups.

## Discussion

HPLC-UV detection method was employed to separate 5-FU from plasma and lymphatic fluid samples. After optimizing the detection conditions, experiments were conducted to optimize the chromatographic separation of the analyses. Good linearity was achieved in the range of 0.5–50 μg/mL, with all coefficients of correlation greater than 0.995. There was no interference under the present analytical conditions of 5-FU in rat plasma and lymphatic fluid (Table 1). The accuracy and precision of the concentrations were all acceptable (Table 2). The results suggested that the analytical method was repeatable and reliable.

Pharmacokinetics is the study of a drug and/or its metabolite kinetics in the body and what the body does to the drugs [26], including chemical taken appropriately into the body (absorption), distributed to the right parts of the body, metabolized in a way that does not instantly remove its activity, and eliminated in a suitable manner [27]. In the current study, the mean extraction recoveries of 5-FU in rat lymphatic fluid and plasma were 56 % and 57%, respectively (Table 1). The original form of 5-FU could be detected in the mesenteric lymphatics as in the plasma with or without RT suggesting that intravenous injection of 5FU can pass from the blood into the lymphatic system.

Here, RT at 2 Gy decreased markedly the AUC of 5-FU by 29% in rats plasma, thus reconfirming the RT-PK phenomena of 5-FU as previously reported [13-15]. The Prior research showed that protein binding of 5-FU in rat plasma was not affected by RT [13]. Additionally, the AUC and MRT of 5-FU in the bile increased while the clearance reduced significantly after RT, suggesting that local RT could facilitate the excretion of 5-FU [15]. Interestingly, the AUC in 5-FU was 4.9-fold greater for lymph than for plasma in the RT group but was 3.3-fold in the non-RT group (*p* = 0.001), however, the concentration of 5-FU in lymphatic fluid and AUC in RT group was not different from that in the control group (Figure 2). Considering the parameters of PK in the plasma of 5-FU, there was not significant different in Vss but with higher CL in RT group (Table 3). These results suggest that the metabolism of 5-FU might be changed by RT and the distribution from blood circulation to the tissues might not be changed.

Radiotherapy may inevitably damage normal tissue and impair the vascular and lymphatic systems, thus causing endothelial cell loss [17] and hypertrophy of surviving endothelial cells [28], which has been associated with enhanced vascular permeability. Radiation-induced increase in vascular permeability is dose dependent between 5- and 20-Gy single doses [29]. It is known that ionizing radiation commonly increases capillary permeability to fluids even within one hour after irradiation [16]. However, much less is understood about the influence of daily RT dose for antineoplastic agents in the lymphatic system. Modification of chemotherapeutic formulation to increase the lymphatic exposure could be a strategy to modulate drug efficacy [30]. For example, increase in docetaxel concentration in circulation and decrease in such concentration t in mesenteric lymph node were accompanied with enhanced anti-tumor efficacy [31]. However, the biological meaning of drug concentration detected in visible gross lymph nodes may differ from that detected in identifiable lymphatic vessels. Little is known about the volume of lymphatic fluid pool and the amount of drug distribution into the lymphatic system after administration [32]. This uncertainty combined with our previous finding of abscopal effect of RT on 5-FU PK may have some impact on clinical practice of CCRT in a statue out of current knowledge level and merits further scientific investigation. In the current study, the parameters of PK of 5-FU in lymphatic fluid were no differences between with or without RT groups (Table 3). It hints that single daily RT dose would not change the permeability of vessel or lymphatic system in delivery of 5-FU.

Compared with surgery alone, neoadjuvant or adjuvant RT for locally advanced rectal cancer could reduce the risk of local recurrent [33]. However, adjuvant CCRT for T3 or T4 or node-positive rectal cancer patients had better recurrent-free survival, overall survival and lower local recurrent rates than adjuvant RT alone [8]. Similarly, neoadjuvant CCRT also achieves lower rates of recurrence than RT alone [34]. The lymphatic system is a chief component of the immune system and acts as a secondary circulation system to drain excess fluids, proteins and waste products from the extracellular space into the vascular system [35]. The regional lymph nodes, once invaded by tumor cells, act as reservoirs where cancer cells take root and seed into other parts of the body [36-39]. The metabolism of 5-FU might be modulated by RT and the distribution from blood circulation to the tissues might not be changed. Additionally, 5-FU can pass through into the lymphatic system. The current analysis sheds light on ambiguities of prior data and may be useful for explaining why CCRT had better locoregional control rates than RT alone in either neoadjuvant or adjuvant setting for locally advanced rectal cancer patients.

The liver catabolyzes about 80% of 5-FU via the dihydropyrimidine dehydrogenase (DPD) pathway, a rate limiting step in the catabolism of 5-FU [40], to generate toxic 5-fluoro-5,6-dihydro-uracil (5-FDHU) [41]. The anabolic pathway, via orotate phosphoribosyl transferase (OPRT), produces active metabolites including 5-fluorouridine- 5’-monophosphate (FUMP), 5-fluorouridine (5-FUrd), and 5-fluoro-2'-deoxyuridine (5-FdUrd) [42]. The severity of adverse events was associated with increased 5-FU/5-FDHU AUC ratio [43]. In the current study, the AUC of 5-FU in the plasma after RT was decreased. Further, the metabolism of 5-FU might be modulated by RT but not the distribution from blood circulation to the tissues. It is worth to investigate the effects of RT on the metabolism of 5-FU in the future to provide useful information for therapeutic drug monitoring of 5-FU to reduce the risk of developing severe toxicities after drug administration concurrent with radiotherapy.

## Conclusion

To our best knowledge, this is the first study proving that local irradiation can significantly modulate the systemic pharmacokinetics of 5-FU. The metabolism of 5-FU might be modulated by RT and the distribution from blood circulation to the tissues might not be changed. This study may provide an experimental clue to understanding the unexplained biological enhancement of antineoplastic agents in the era of pelvic CCRT for improving locoregional control of locally advanced rectal cancer patients.
